# The epigenetic regulator CTCF modulates BCL6 in lymphoma

**DOI:** 10.18632/oncoscience.239

**Published:** 2015-09-14

**Authors:** Ana Batlle-López, María G. Cortiguera, M. Dolores Delgado

**Affiliations:** Instituto de Biomedicina y Biotecnología de Cantabria, IBBTEC (CSIC-Universidad de Cantabria) Santander, Spain

**Keywords:** BCL6, lymphoma, CTCF, epigenetics

Despite the improvements in the diagnosis and in the approaches to treatment, aggressive lymphomas are still an important cause of morbidity and mortality. There is, therefore, considerable interest in understanding the mechanisms involved in the process of lymphomagenesis. Germinal centers are dynamic structures within the lymph nodes containing highly proliferating B-cells that are undergoing mutations, in order to produce high affinity maturation antibodies, and for these reasons are predisposed to the formation of malignancies. Indeed, a number of mature lymphoid neoplasms are generated in these structures.

BCL6 is an important transcriptional repressor known to be the master regulator of the germinal center, required to differentiate follicular B cells into germinal center B cells and to maintain these cells in this state [1]. *BCL6* is highly regulated during B cell differentiation such that naïve B-cells and terminally differentiated plasma cells do not express the protein whilst germinal centre B-cells expresses large amounts. BCL6 deregulated expression is sufficient to induce a lymphoproliferative disease similar to the human Diffuse large B-cell lymphoma in mice. Therefore, is of critical importance to understand how the expression of BCL6 is regulated. However, many aspects related to the mechanisms involved in the regulation of the gene in normal B-cells and in lymphomas derived from germinal center B-cell still remain unclear. Our recent study sheds light on the epigenetic regulation of *BCL6* expression by the CTCF chromatin regulator [2].

CTCF is a highly conserved, ubiquitously expressed zinc finger protein. The functions described for CTCF include transcriptional activation or repression, insulator binding protein and global organization of chromatin, through the facilitation of long-range interactions and chromatin looping [3]. CTCF is involved in the regulation of various genes implicated in cancer, frequently through epigenetic mechanisms. Chromatin structure and modifications are considered nowadays critical aspects of gene expression regulation. The main epigenetic mechanisms are histone modifications as acetylation or methylation, and DNA methylation in CpG dinucleotides. Gene activation is related with unmethylated DNA near the gene promoters and with marks of active chromatin such as histone acetylation or histone methylation in specific residues. These modifications confer an open chromatin structure at that locus. On the contrary, gene repression is related with specific repressive histone marks that recruit repressive complexes, giving rise to a close conformation of the chromatin and silencing of gene expression [4].

**Figure 1:**
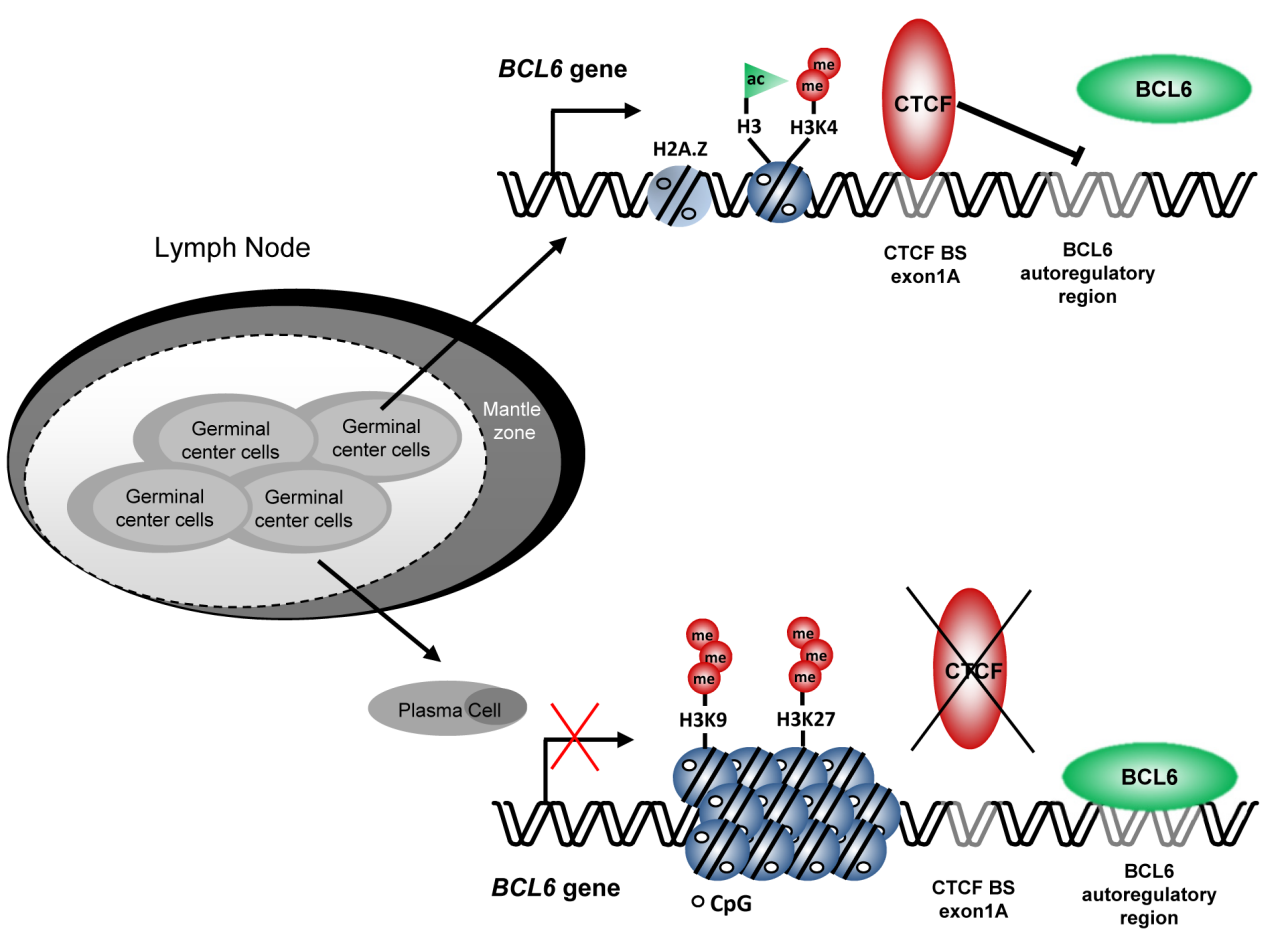
Model for BCL6 regulation by CTCF CTCF binding to exon1A, associated with active histone marks, impairs BCL6 negative autoregulation allowing BCL6 expression in germinal center B cells. For differentiation into plasmatic cells, CTCF is released from exon1A site allowing BCL6 negative autoregulatory circuit to function. This leads to recruitment of repressive marks, chromatin condensation and inhibition of BCL6 expression. In turn, BCL6 downregulation allows *BLIMP1* and *IRF4* expression, both required for plasma cell differentiation.

In our work we show that CTCF binds to a previously undescribed site in *BCL6* exon1A [2]. Features that make CTCF binding to exon 1A a likely mechanism to induce *BCL6* expression in germinal center cells are: i) CTCF binding to this site was associated with high levels of BCL6 expression, while lack of CTCF binding was correlated with reduction of *BCL6* expression in germinal center B-cells and induction of plasma cell markers; ii) CTCF binding was required to maintain *BCL6* expression in germinal center cells by avoiding the well-known *BCL6* negative autoregulation; iii) CTCF binding to *BCL6* exon1A was associated with presence of active histone marks and protection against repressive marks. These findings are summarized in the Figure 1. Notably, CTCF has also been associated to negative regulation of BCL6 expression upon binding to an unmethylated intron 1 in plasma cells [5]. It is, therefore, tempting to speculate that BCL6 expression might depend on the stage-specific CTCF recruitment to the different *BCL6* binding sites during B lymphocyte development. CTCF binding to exon1A appears to be required to maintain BCL6 expression by protecting BCL6 exon1A from BCL6 auto-repression in germinal center cells. On the contrary, CTCF binding to the intronic region is more likely to be necessary to prevent BCL6 expression in non-germinal center cells and allow differentiation.

BCL6 is an attractive target for therapy and indeed different specific BCL6 inhibitors are currently under investigation. Treatments with drugs that modify the chromatin have enormous potential for cancer therapy [4]. Epigenetic modulation of BCL6 expression might be effective in germinal center derived lymphomas. Preliminary results performed in our laboratory showed that treatment of germinal center derived B cell lines with histone deacetylase inhibitors downregulated BCL6 expression, caused plasma cell differentiation and induced cell death. The possible role of CTCF in this process is currently under study.
